# Gaze in context: non-human eyes can be more salient under ecologically relevant conditions

**DOI:** 10.1017/ehs.2026.10058

**Published:** 2026-06-19

**Authors:** Juan Olvido Perea García, Fumihiro Kano, Marta Sibierska, Vojtěch Fiala, Marta K. Skrok, Dariusz P. Danel, Ewa Katarzyna Ratajczak, Przemyslaw Zywiczynski, Slawomir Wacewicz

**Affiliations:** 1 Center for Language Evolution Studies, Nicolaus Copernicus University, Toruń, Poland; 2 Department of Modern Philology, University of Las Palmas de Gran Canaria (ULPGC), Las Palmas, Spain; 3 Center for the Advanced Study of Collective Behaviour, University of Konstanz, Konstanz, Germany; 4 Max-Planck Institute of Animal Behavior, Konstanz, Germany; 5 Institute for the Advanced Study, Kyushu University, Fukuoka, Japan; 6 Institute of Advanced Studies, Nicolaus Copernicus University in Toruń, Toruń, Poland; 7 Department of Philosophy and History of Science, Faculty of Science, Charles University, Prague, Czechia; 8 Institute of Physics, Faculty of Physics, Astronomy and Informatics, Nicolaus Copernicus University in Toruń, Toruń, Poland; 9 Department of Anthropology, Hirszfeld Institute of Immunology and Experimental Therapy, Polish Academy of Sciences, Wrocław, Poland; 10 Institute of Psychology, Faculty of Philosophy and Social Sciences, Nicolaus Copernicus University in Toruń, Toruń, Poland

**Keywords:** eye-gaze, iris, sclera, communication, colouration

## Abstract

Primate eyes vary strikingly in pigmentation, yet the drivers of said variation are strongly debated. Recent revisions of the cooperative eye hypothesis propose that the human eye’s sclera evolved to enhance gaze communication specifically under challenging conditions of visibility. We tested this idea under ecologically realistic conditions by presenting observers with a live model wearing contact lenses that simulated either a human-like or a chimpanzee-like eye. At a university lab, observers judged gaze direction at different viewing distances and lighting levels. We found no overall difference in efficacy of different eye types. Contrary to expectations, chimpanzee-like eyes outperformed human-like eyes in dim lighting and close-viewing conditions. Human-like eyes yielded the highest accuracy under bright, far-viewing conditions, consistent with a long-distance signalling advantage. Our results demonstrate that ecological visual constraints shape the potential informativeness of distinct ocular configurations. We hypothesize that species-typical eye appearances may be tuned to their species-typical visual ecology.

## Graphical abstract

**Figure:**
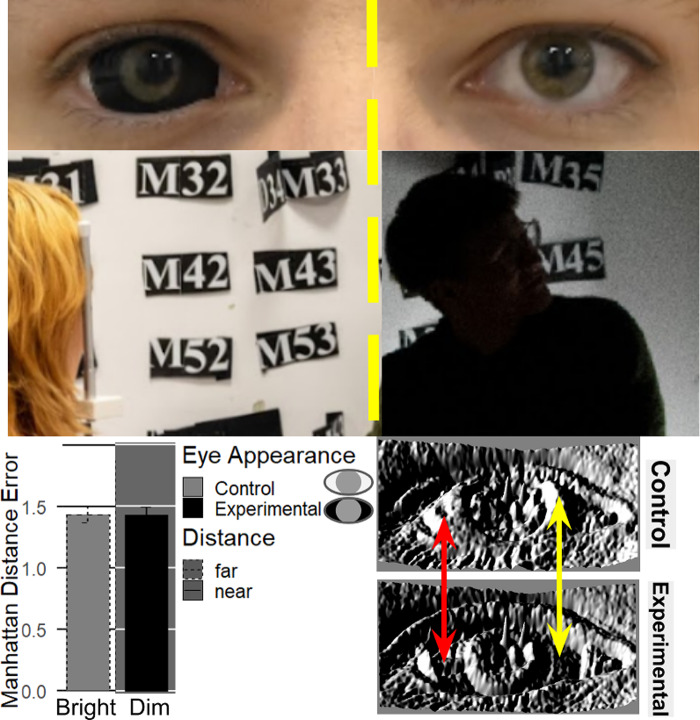

## Social media summary

Primate eyes with dark sclera can be more effective spatial pointers than human eyes depending on distance and lighting.

## Introduction

Primate eyes are remarkably varied in their external appearance (Perea-García et al., 2022), but the selective forces shaping this variation remain contested (Kano, 2023; Perea‐garcía et al., 2025). Early influential accounts emphasized the uniqueness of the human eye within the primate lineage: a horizontally elongated palpebral fissure exposes a large, depigmented sclera, whose sharp contrast with a darker iris was proposed to facilitate communication via gaze. This idea, formalized as the gaze-signalling hypothesis (Kobayashi & Kohshima, 1997, 2001) and later expanded into the cooperative eye hypothesis (CEH) (Tomasello et al., 2007), posits that conspicuous human eyes underpin capacities such as joint attention and large-scale cooperation, specifically in close-range interactions.

Recent studies, however, have challenged the assumption of human uniqueness in form and function. Quantitative examinations show that non-human primates may also display conspicuous eye morphologies (Perea-García et al., 2019; but cf. Caspar et al., 2021; Mearing & Koops, 2021). Crucially, conspicuousness can arise through different luminance configurations: in humans and bonobos, the iris is typically darker than a depigmented sclera (Type 1: Perea-García et al., 2019), whereas in chimpanzees and other species, the sclera is darker and surrounds a lighter iris (Type 2: Perea-García et al., 2019). Image-based simulations suggest that both types remain visible across ecologically relevant distances (Whitham et al., 2022a, 2022b). These studies supported the idea that conspicuousness is not unique to humans but can be attained by alternative ocular configurations.

Following these results showing that human and non-human primate eyes are comparably suited for referential functions, more recent studies argue that Type 1 eyes enhance gaze perception specifically under visually challenging conditions. Yorzinski and Miller (2020) found that darkening the sclera to resemble Type 2 eyes slowed gaze-direction judgements, especially with small or inverted faces. In a related experiment, Yorzinski et al. (2021) manipulated stimuli to remove iris–sclera contrast under simulated day- and night-time lighting; participants were consistently faster and more accurate with Type 1 sclerae. These authors concluded that depigmented sclerae may have evolved to improve gaze perception across lighting regimes, potentially supporting activities such as nocturnal group hunting.

Kano et al. (2022b) used image analyses and visibility simulations showing that human gaze direction is not uniquely visible at close range in bright light but becomes substantially more visible than great-ape gaze under distance and dim illumination. In a complementary behavioural experiment, Kano et al. (2022a) tested human and chimpanzee observers judging eye direction from Type 1 versus Type 2 stimuli under simulated low visibility (reduced brightness, smaller apparent size). Both species performed more accurately with Type 1 eyes, and this advantage increased as visibility decreased. Kano et al. (2022a) interpreted these results as evidence that the human eye morphology offers a specific advantage under degraded visual conditions. Together, these studies form a revised version of the CEH in which the superiority of human-like eyes emerges primarily under low illumination or greater viewing distances (Kano, 2023).

The control gained in computerized tests of Type 1 and Type 2 eyes under simulated low-light and long-distance conditions necessitated a degree of loss of fidelity in additional cues important in natural eye-gaze perception. Dynamic cues such as micro-movements or subtle head adjustments were absent, even though motion is known to mitigate effects of polarity inversion (Anderson et al., 2016). Corneal reflections, shown to influence judgements of gaze direction (Anstis, 2018), were either deleted (Yorzinski & Miller, 2020; Yorzinski et al., 2021) or inverted (Kano et al., 2022a). Furthermore, in order to isolate the effect of contrast polarity, Type 2 appearances were simplified, for example, by uniformly darkening the portions of the sclera most distal to the iris (e.g. Kano et al., 2022a). Lastly, these studies employed screens, which, as stated by Yorzinski et al. (2021), limit the generalization of the results to actual conditions of luminosity. Furthermore, recent evidence in psychophysics demonstrates that eye-gaze perception differs systematically between two-dimensional (2D) and three-dimensional (3D) stimuli, suggesting that previously reported effects stem from distortions of spatial cues (Horstmann & Linke, 2025). Taken together, these findings highlight the need for experimental designs that preserve a fuller range of perceptual cues when testing the visibility of different eye morphologies, especially under visually challenging conditions.

Primate species inhabit environments that differ dramatically in ambient illumination. From very bright open savannas in direct sunlight to very dim tropical forests (Dominy & Melin, 2020): above the canopy, illumination can exceed 10,000 lux, whereas dense foliage may reduce midday light to 100–200 lux at the forest floor. Overcast skies can reduce illumination by around 0.4 log units, so at the forest floor under a dense canopy, illumination can vary widely from 5–10 lux to more than 1,000 lux, depending on the time of day. These ecological conditions encompass several recurrent scenarios in which gaze communication might take place: shaded forest understories, overcast days in open habitats, and twilight transitions between day and night. At least one previous study testing outdoor lighting demonstrates drastic effects of illumination on human eye-gaze visibility (Fotios et al., 2015), although their lighting conditions (0.01–1 cd/m^2^) were less relevant to diurnal primates. These findings underline the importance of situating eye-gaze perception within the variable light regimes that primates naturally encounter.

Here, we directly test the revised premises of the CEH by manipulating eye appearance, viewing distance, and lighting conditions in a live-model paradigm. Our design retains the three-dimensionality and reflective properties that earlier studies traded for experimental control, and it more closely resembles the natural distribution of luminance contrast and spatial cues of real non-human primate eyeballs, including local variation in scleral pigmentation. Lastly, our lighting regimes are informed by and resemble actual primate visual ecology.

## Materials and methods

### Preregistration

This study was preregistered on AsPredicted (#199907).

### Participants

Ethics approval was obtained from the Research Ethics Committee of the Faculty of Philosophy and Social Sciences, Nicolaus Copernicus University in Toruń (Permit 31/2024). Twenty adult participants (*N* = 20; *F* = 15, age = 26.45 ± 6.04) took part in the study. Participants self-described as ethnically Polish and could thus be described as WEIRD (Western, Educated, Industrialized, Rich, Democratic: Heinrich et al., 2010). These were mostly university students, especially tied to Social Science and Humanities – hence the skewed sex ratio. We do not believe this skew could have influenced our results in any meaningful way – we are testing the visibility of different eye configurations, assuming that there is a deeply conserved visual system between contemporary and ancestral humans; differences between sexes of the same species should not be of consequence. Although our screening criteria specified that participants should have normal or corrected-to-normal vision, we tested visual acuity and asked for self-reported colour-blindness. Visual acuity was measured using an 8-position Landolt ‘C’ chart at 3 m (Good-Lite Co., Elgin, IL, USA). Participants were first tested under dim illumination to reduce memorization effects before testing under bright light. Under bright illumination, logMAR scores ranged from −0.28 to 0.25 (mean = −0.028, SD = 0.149). Most participants (nine out of the sample) scored between −0.10 and 0.10 logMAR, indicating generally high acuity, while a single participant had a lower acuity of 0.25 logMAR. Under dim illumination, logMAR scores ranged from −0.097 to 0.708 (mean = 0.202, SD = 0.207). Performance decreased compared with the bright condition, with seven participants scoring above 0.30 logMAR and one participant showing the lowest acuity of 0.708 logMAR. Because 0.708 logMAR was well below the acuity of other participants, we compared this participant’s error rate with the average of the sample in the dim condition. The participant with the worst acuity made 169 errors, which was well below the average of the rest of participants in the same condition (mean = 217). Thus, we kept the data from said participant in the analyses. No participants declared being colour-blind. Participants were exposed to all combinations of conditions, which were randomized. Participants took part in two sessions – in one of them, participants went through all the tasks with Type 1 eye appearance (no lenses), and in the other, they went through all the conditions with Type 2 eye appearance (via black scleral lenses). The order in which they were exposed to Type 1 or Type 2 eye tasks was counterbalanced across all participants. One participant attended only the first session, so another participant was recruited to reach the intended *N* = 20.>>>

### Apparatus and stimuli

The task took place under controlled indoor conditions. The live model sat opposite each participant at a distance of either 130 cm (near distance) or 530 cm (far distance). These distances were employed to maximize the space available in the testing room. Each distance approximates peripersonal and extrapersonal space in humans (<160 and >160 cm, respectively: Stone et al., 2018), thus approximating distinct situations in primate ecology and sociality.

For each distance, there was a separate 7 × 10 grid at which the live model gazed in each trial. These two grids had marked points to be looked at by the model. The points in the grids were placed on two contiguous walls behind the participant. The walls met at 90°. Horizontal spacing of points in both grids was by 4.5° of visual angle from the position of the live model in each distance condition. Both grids were concentric, such that some points in each grid overlapped. Thus, referents were labelled M (from Polish ‘mała’; small) for the near and D (from Polish ‘duża’; large) for the far conditions, together with a unique identifying number (1–70). On perfectly overlapping referents, they were tilted slightly so both were visible to the participant. Horizontal spacing was decided on the basis of several factors. First, we piloted the most extreme gazing distance at which the live model could stare for a sustained amount of time without feeling discomfort, spanning an arc of 45°. Then, we piloted various spacing distances of the grid until we found intervals that led to at least some amount of error when guessing the intended referent of the live model without lenses. Vertical spacing was fixed at ∼4°. This was decided on the basis of the distance between the live model’s eyes and the floor (128 cm). This distance was doubled to place the topmost row, to keep the live model’s gaze at the center of the grids. Vertical intervals were then again decided on the basis of piloting with Type 1 eye appearance, to attain at least a minimum of errors. The central row of both grids was set at 128 cm. Crucially, visual (eyeball rotational) angles were kept constant in both distance conditions. Spacing between referents was decided to well above previous estimations of the thresholds for detectability of eye rotation (1°: Vida & Maurer, 2012; above 3° for almost certain identification: Anderson et al., 2011). Thus, differences between distance conditions can only be attributed to distance, and not differences in visual angle. The live model used a chinrest (Reha0119, Hasomed GmbH, Paul-Ecke-Str. 1, 39114 Magdeburg/Germany) to stabilize her head, thus minimizing the influence of head direction. The chinrest was set so that the middle row of both grids corresponded with the distance of the live model’s eyes at 90° from the floor. The live model was instructed to keep their head direction as still as possible. We did not record the live model’s face to ensure that there was absolutely no movement. However, during pilot sessions, there were no noticeable movements.

**Figure 1.**
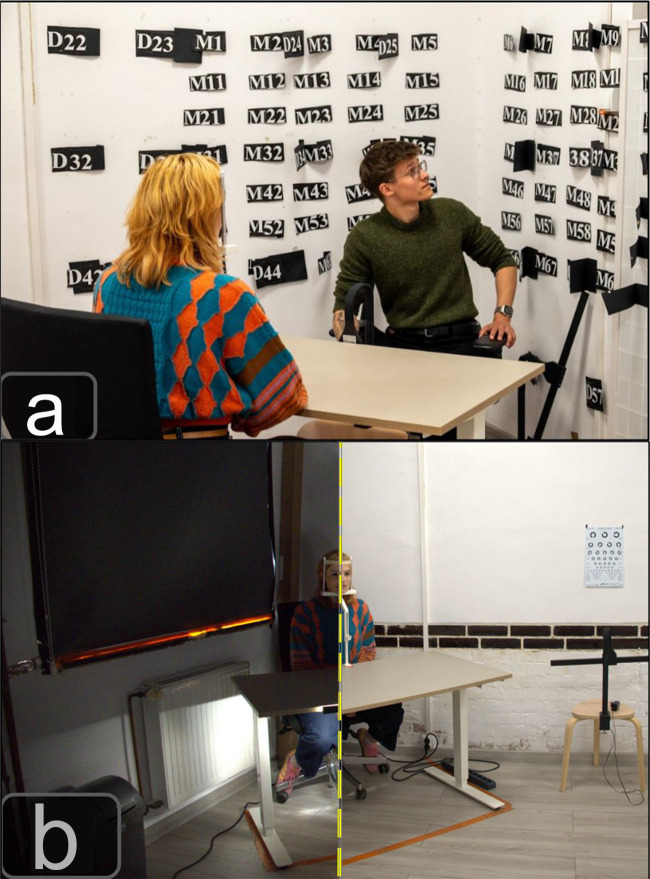
(a) Representation of the ‘near’ condition with bright lighting. In the ‘near’ condition, the live model (red-blue top) gazed at the small grid, marked with ‘M’ behind the participant (black shirt). Points in the grid with ‘D’ were used for the ‘far’ condition. (b) Representation of the view of the live model in the ‘far’ condition with dim (left) and bright (right) conditions. Figure 1 long description.

Participants sat on a swivelling stool, allowing them to look behind at the intended marks on the wall (see Fig. 1). A detailed representation of the physical layout to facilitate replication can be found in the Supplementary material (S1).

Lighting conditions were manipulated via artificial diffuse lighting. Light-blocking blinds were installed on the windows to prevent any natural lighting from entering the room. For the bright condition, the ceiling lights were turned on. These consisted of four panels with diffusers. Photometer readings throughout the room ranged from 400 to 550 lux (Sekonic L-308X-U FLASHMATE Light Meter). This falls within the photopic range typical of the low end of daylight environments experienced by diurnal primates, where visual acuity and cone-mediated colour discrimination are both fully functional. This range also matches illuminances commonly encountered in well-lit indoor human environments. For the dim condition, we turned off all ceiling lights and turned on dimmable desk LED lamps (*MatMay* brand), set to the lowest level. The lamps were carefully angled so that referents on the wall were visible, but lux readings remained between 3 and 10 when performed right beside the participant’s and live model’s eye region. The dim condition thus approximates the ecological lighting typical of civil twilight (just after sunset or before sunrise) or heavily shaded overcast forest environments (Dominy & Melin, 2020). For the dim condition, we used the highest temperature in the lamps (6000 K) as it most closely resembled the white lights in the lab ceiling used in the bright condition. This choice was pragmatic rather than intended to most closely align with ecological conditions. A detailed description of the lighting set-up to facilitate replication can be found in the Supplementary material (S2).

**Figure 2.**
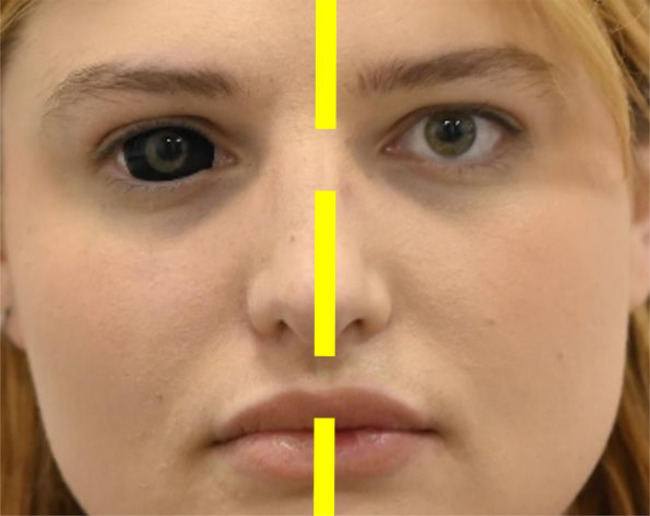
Side-by-side comparison of the two eye appearances (Type 2 on the left, Type 1 on the right) used in the study.

Eye appearance was manipulated using commercial scleral contact lenses (ColourVUE *Dark Angel* 22 mm model; https://colourvue-lens.com), which rendered the sclerae uniformly black while preserving iris visibility. Thus, in the experimental condition, the live model’s natural light irises were surrounded by the dark scleral lenses (resembling Type 2 eye appearances). Importantly, the portions of the anterior eye most distal to the iris remained uncovered by the scleral lenses, resembling the typical appearance of primate species with pigmented eyeballs, in which pigmentation fans out away from the limbus, becoming lighter in the portions of the eyeball most distal to the iris (cf. brightness measurements of conjunctiva vs. sclera in Perea-García et al., 2022; APIT vs. PPIT in Perea-García et al., 2024; Anterior Peri Iridal Tissues vs. Posterior Peri Iridal Tissues in Perea-García et al., 2024). For the Type 1 appearance, the live model wore no lenses, so her irises were surrounded by naturally depigmented conjunctiva and sclera (Type 1). Although the design would have been fundamentally stronger if the live model had used transparent contact lenses for the control condition, we opted to avoid ‘control’ lenses in order to minimize discomfort for our live model resulting from the usage of lenses for extended periods of time. At the same time, we see no plausible mechanism that could meaningfully affect our results. The live model was blind to the hypotheses. The live model was not recorded, but the researchers who collected the data did not observe any systematic difference in behaviour across conditions. The stool where participants sat was adjusted in height for each participant, so that their eyes were at the same height as those of the live model. The live model was instructed not to wear make-up and to wear the same top during experimental sessions to avoid possible confounds. For comfort and stability, the live model kept her hands on the table surface throughout the experiment. We decided to keep a single live model as a stimulus to minimize variability due to differences in the stimuli. Nonetheless, recent evidence shows that most variability is due to intrinsic properties of the observer, and not the stimulus (Alting & Horstmann, 2025). Photographs of the live model with and without lenses can be seen in Fig. 2.

### Procedure

The participants were informed that their task consisted of determining where the live model was looking on the grid behind them. Before each trial, the live model consulted a randomized list of target positions, fixated on the intended referent, and tapped a pen on the table to signal the start of the trial. The live model kept their gaze on that referent for the duration of the trial. Participants reported the location they believed the live model was gazing at via a mobile questionnaire form. No accuracy feedback was provided to minimize learning effects. To ensure comprehension of the task by the participants, each session began with three mock trials using extreme grid locations. Each participant completed two sessions (Type 1 and Type 2 eye appearance), each consisting of four blocks (near-bright, near-dim, far-bright, far-dim), each encompassing 30 trials. This resulted in a total of 240 trials per participant, and 4,800 overall data points for the whole group. This differs from the preregistered number of trials, in which we proposed 50 trials per block. The change was implemented to reduce fatigue for the participants and decrease exposure time to the lenses for the live model. Session order was counterbalanced. Within sessions, blocks were randomized.

### Design and analyses

The dependent variable was gaze error, calculated as the *Manhattan distance* – that is, the sum of the horizontal and vertical steps – between the grid point the live model was gazing at and the point selected by the participant. This metric captures how far off a response was in terms of grid units, treating movement across the grid like navigating a city block (i.e. without diagonal shortcuts). Using Euclidean distances or Manhattan distances in equally spaced referents would be numerically equivalent. Euclidean distances would have given a false impression of a continuous measure. We used Manhattan distance because errors were scored as displacement across discrete spatial positions, rather than as continuous physical distances in centimetres. Using irregularly spaced referents across sessions would have been unfeasibly demanding; comparability across sessions would be reduced, and interpretability would be compromised. Statistically, this could have also lowered the power of our sample by including new factors to account for (e.g. block-level random errors). The model included fixed effects and interactions between lighting (bright/dim), distance (near/far), and eye appearance (Type 1/Type 2). Our preregistered analysis plan proposed a Bayesian GLMM with log link and negative binomial error structure if overdispersed, using the brm() function of the brms package in R (Bürkner, 2017). We slightly deviated from this plan; we used a zero-inflated negative binomial model to accommodate the high frequency of perfect responses (i.e. zero error) and to resolve convergence issues encountered without the zero-inflation component, which also reduced computational demands. We allowed the intercept to vary by session nested within participants. As indicated in the preregistration plan, we did not include trial as a random effect because we observed no training effect (output from the model in Table S3 in the Supplementary material). The final model formula was thus: Error ∼ light × distance × eye appearance + (1 | participant/session). All analyses were conducted using R version 4.4.2.

## Results

### Main study

The main model converged well, with R-hat = 1.00 for all parameters, effective sample sizes over 1,000, and no evidence of residual autocorrelation. Posterior predictive checks indicated a good fit for the data. Chains mixed well, with a ‘fuzzy caterpillar’ appearance.

**Figure 3.**
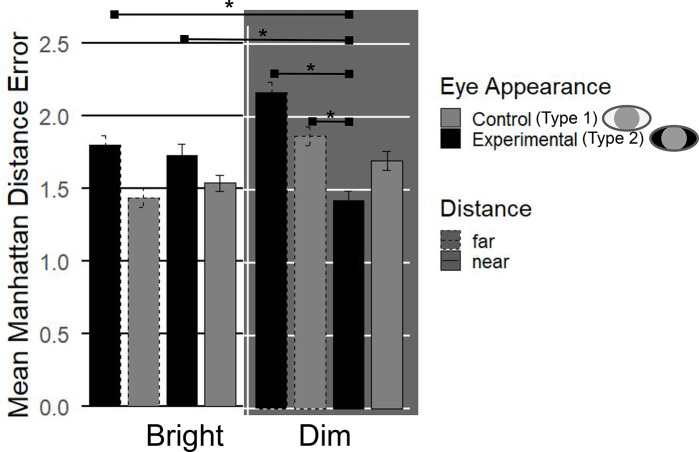
Mean Manhattan error for all combinations of light, distance, and eye appearance. Grey bars represent the control eye appearance (Type 1), black bars represent the experimental eye appearance (Type 2). Bright and dark backgrounds represent the ‘bright’ and ‘dim’ conditions, respectively. Figure 3 long description.

*Notes*: Continuous and dashed lines represent the near and far conditions, respectively. Credible differences are indicated with *. Whiskers depict SE.

All estimates represent log-linear coefficients (*β*) for the expected error count (in Manhattan distance). A value of *β* > 0 indicates increased expected error; *β* < 0 indicates reduced error. Exponentiated values of *β* reflect multiplicative changes in expected error. In Bayesian statistics, ‘credible differences’ are the closest analogue to frequentist ‘significant differences’. They are typically assessed by comparing the posterior distributions of conditions; differences are considered credible when the CrI for each condition do not overlap, indicating substantial probability that the effect is non-zero. Error was significantly greater in the dim condition compared to the bright condition (*β* = 0.26, 95% credible intervals, CrI, [0.17, 0.35]), corresponding to an estimated 30% increase in expected error (exp(0.26) ≈ 1.30). There was no strong evidence of distance, by itself (*β* = 0.07, 95% CrI [−0.03, 0.16]); on the response scale, which amounted to a ∼7% increase compared to the far condition (but the interval included zero). Participants made more errors when viewing the Type 2 eye appearance (*β* = 0.24, 95% CrI [0.09, 0.38]), leading to a ∼27% increase in expected error. Critically, a significant three-way interaction was observed between light, distance, and eye appearance (*β* = −0.21, 95% CrI [−0.39, −0.04]). Mean error by combination of factors is summarized in Table 1. Post hoc, we also tested pairwise comparisons using the emmeans package in R (Lenth, 2025) to extract estimated marginal means from the fitted Bayesian model. All combinations of light, distance, and eye appearance were compared using the pairs() function without multiple comparison correction. Credible differences were determined by whether the 95% highest posterior density (HPD) interval excluded zero. Because previous studies concluded that Type 2 appearances would be less effective to convey signals in challenging visual conditions, we filtered the comparisons to identify which combinations of factors differed significantly from the *dim near Type 2* combination (Table 2). All conditions with credible comparisons were significantly greater in errors (Fig. 3).

**Table 1.** Mean Manhattan error and standard deviation by combination of factors Table 1 long description.

Condition	Mean error	SD
Dim near Type 2	1.43	1.50
Bright far Type 1	1.44	1.60
Bright near Type 1	1.54	1.35
Dim near Type 1	1.70	1.62
Bright near Type 2	1.73	1.83
Bright far Type 2	1.80	1.65
Dim far Type 1	1.87	1.61
Dim far Type 2	2.16	1.81

**Table 2.** Pairwise comparisons with the dim, near, and experimental conditions Table 2 long description.

Comparison	Estimate (log)	Exp (estimate)	Lower HPD	Upper HPD	Credible	Direction
Bright near Type 2	0.1903	1.21	0.1012	0.2804	Yes	Greater
Bright far Type 2	0.2268	1.26	0.1371	0.3169	Yes	Greater
Dim far Type 1	0.2562	1.29	0.1061	0.4025	Yes	Greater
Dim far Type 2	0.4146	1.51	0.3263	0.4996	Yes	Greater
Bright far Type 1	−0.0087	0.99	−0.1692	0.1391	No	Less
Bright near Type 1	0.0573	1.06	−0.0917	0.214	No	Greater
Dim near Type 1	0.1582	1.17	−0.0028	0.3016	No	Greater

### Referent-centrality analyses

Although this analysis was not part of the preregistered plan, visual inspection of participants’ performance across the grid (see heatmap in Supplementary material S4) suggested a potential effect of referent centrality – that is, the visual angle of the target referent – on performance. To formally assess this, we classified referents as central or peripheral. Peripheral referents were those at least three Manhattan distance away from the two most central ones. We then included referent centrality (central vs. peripheral) as an additional factor in the model (Error ∼ light × distance × eye_appearance + centrality + (1 | participant/session). The results showed that errors were higher under dim lighting (*β* = 0.26, 95% CrI [0.18, 0.35]) and for the Type 2 eye appearance (*β* = 0.24, 95% CrI [0.08, 0.39]); performance was not reliably affected by viewing distance alone (*β* = 0.06, 95% CrI [−0.03, 0.15]). Critically, a three-way interaction persisted between lighting, distance, and eye appearance (*β* = −0.23, 95% CrI [−0.40, −0.05]). Finally, participants made fewer errors when the referent was peripheral than when it was central (*β* = −0.19, 95% CrI [−0.23, −0.14]). Further, visual inspection of mean Manhattan distance error by referent centrality (Figure S5) suggested that the advantage of the Type 2 stimulus in the *dim near* combination of factors was greater in peripheral referents.

To compare our results with psychophysics studies of similar scope, we conducted additional analyses exploring gaze estimation biases. Consistent with previous reports by Alting and Horstmann (2025) and Horstmann and Linke (2025), participants generally overestimated gaze direction, particularly for horizontal orientations and near distances (Figure S6). Notably, because this bias occurred even with our 3D model stimulus, it cannot be attributed to a depth-perception bias. Interestingly, at far-peripheral referents – especially at the corner referents of the 7 × 10 grid – participants tended to underestimate gaze direction, a pattern not documented in previous studies.

### Visual discriminability analyses

Our results indicate that participants performed better with the experimental eye (Type 2) compared to the control eye (Type 1), particularly under dim lighting conditions. This was unexpected, given the predictions of recent revisions of the CEH (e.g. Kano, 2023). To explain our results, we modelled the visibility of the live model’s eyes when depicting Type 1 and Type 2 appearances. These two appearances differ in reflectance around the iris (sclera vs. scleral lens), while retaining the same reflectance in the iris. Additionally, our Type 2 stimulus included additional contrasts such as between the scleral lens and the underlying sclera and could have increased the local contrast between the ocular area and surrounding skin (due to enhanced contrast between the scleral lens and skin). To estimate visibility, we modelled Type 1 and Type 2 stimuli under a luminance contrast-sensitivity function (CSF; Westland et al., 2006; based on Barten, 1999). A full description of the methods to estimate visibility in each combination of factors can be found in the Supplementary material (S7). Our resulting models (Figure S9) suggested, specifically with regard to the *dim near* combination of factors, that the pupil and iris were visible in both eye appearances, and both the scleral lens and natural sclera contrasted with the surrounding skin. In addition, the scleral lens created a contrast with the underlying sclera that was absent in the Type 1 stimulus.

## Discussion

Our results add to the recent literature showing that Type 2 eyes can be as effective for conveying referential information as Type 1 eyes. Specifically, errors were lowest with Type 2 eyes under dim light at close range, whereas errors were lowest with Type 1 eyes under bright light at longer range. These context-dependent results suggest that different morphologies may be suited to different lighting regimes, consistent with differences in the visual conditions under which gaze signalling operates (e.g. closer range and more shaded for primates with Type 2 eyes; longer range and brighter for primates with Type 1 eyes). This finding is not consistent with the original CEH, according to which the human eye constitutes an appearance whose ‘gaze direction [is] easier for others to follow across all contexts’ (Tomasello et al., 2007), nor with revisions of the CEH proposing that human eyes confer advantages under visually challenging conditions (Kano, 2023; Kano et al., 2022b, 2022a; Yorzinski et al., 2021). Instead, these findings suggest that the perceptual salience of eye features is context-sensitive, rather than fixed across viewing conditions. This does not imply a simple or deterministic mapping between species-level ecologies and eye morphology. Eye appearance is likely shaped by multiple interacting constraints, including photo-regulatory demands, physiological factors (e.g. the presence or absence of epithelial stem cells in the lateral limbus: Grieve et al., 2015), reliance on ocular signalling (not limited to gaze following), and visual conditions.

The idea that human-like eye appearance excels for signalling under visually challenging conditions may rely on assumptions about environments that do not reflect the ecology of diurnal primates (Yorzinski & Miller, 2020). Moreover, substantial within-species variation in peri-iridal pigmentation in great apes (Clark et al., 2023; Kano, 2023; Perea‐García et al., 2025) is inconsistent with strong directional selection towards a single optimal configuration. Rather than diminishing the importance of gaze, these findings show that conspicuity alone is neither necessary nor sufficient to explain the evolution of primate eyes.

These findings inform long-standing accounts of the influence of luminance polarity in eye-gaze perception. Classic polarity-reversal studies proposed an ‘expert system’ heuristic in which observers rely on the darker region to infer gaze direction (Ricciardelli et al., 2000). Later work argued that the human-typical dark-on-light configuration (Type 1) generally enhances gaze communication, with both humans and chimpanzees judging gaze direction more accurately from Type 1 than Type 2 eyes (Kano et al., 2022a), a pattern linked to clearer eye and iris outlines. Perceptual asymmetries for dark-on-light contrasts may further amplify this advantage (Lu & Sperling, 2012). However, studies using more naturalistic stimuli have shown that polarity alone cannot account for gaze perception and instead support a multiple-cue account in which luminance interacts with motion, reflections, and 3D structure (Anderson et al., 2016). Our findings qualify both the ‘expert system’ and ‘multiple-cue’ accounts. The bright-far results for Type 1 eyes are consistent with revised CEH proposals that human eyes remain visible under challenging conditions (Kano et al., 2022a; Yorzinski & Miller, 2020; Yorzinski et al., 2021), especially due to distance (Kano, 2023). Yet the strong advantage of Type 2 eyes in dim-near conditions contradicts assumptions of unconditional Type 1 superiority (Ricciardelli et al., 2000) and diverges from claims that human-like eyes outperform others under low visibility (Kano et al., 2022a; Yorzinski et al., 2021). Substantial differences in our design preclude direct comparisons with previous studies. These results are, nonetheless, inconsistent with the view that features such as positive contrast polarity or scleral depigmentation provide general-purpose advantages.

Our visibility analyses explain the differences in performance for Type 1 and Type 2 appearances across visual contexts. The scleral lens in Type 2 eyes introduced additional contrasts between the lens and underlying sclera and between the eye region and skin. These may have been particularly informative for peripheral referents and likely drove the Type 2 advantage under dim, near-viewing conditions. Spatial-frequency considerations help explain the complementary Type 1 advantage at distance. Human-like eyes contain strong high-, mid-, and low-frequency components contributing to gaze visibility (Vida & Maurer, 2015) and maintain their structure across distance (Kano et al., 2022b). Distance degrades high- and mid-frequency information (Goldstein, 2010), and dim light reduces amplitude across all frequencies. While Type 2 eyes contain all frequency components, their gaze-relevant cues depend more on fine spatial detail, making them more vulnerable to distance-based degradation. This explains the reduced performance of Type 2 eyes compared to Type 1 eyes under far-viewing conditions. Some discrepancies between modelled visibility and observed performance indicate that cues beyond simple luminance contrasts contributed to performance. Although CSF models suggested that pupil-iris, iris–sclera, and sclera-skin contrasts in the Type 1 eye should be below threshold in the dim-far condition, participants nonetheless performed better with Type 1 eyes there. This suggests contributions from outlines, eyelashes, slight eye movements (Anderson et al., 2016), and conjunctival reflections (Anstis, 2018). Whenever the outline of th eye is not under control, differences in scleral exposure should also be considered (Boyer-Brossseau et al., 2025). Gaze perception thus relies on a constellation of features, not a single cue.

Our results extend recent psychophysical findings on gaze-estimation biases. As in Horstmann and Alting and Horstmann (2025) and Horstmann and Linke (2025), we observed robust horizontal overestimation that increased at closer viewing distances. The magnitude of these biases fell between their 2D and 3D conditions, likely due to geometric ambiguities introduced by our set-up: referents were located behind observers, and the room’s 90° geometry removed planar distortions while adding spatial uncertainty. We also observed a previously unreported pattern – systematic underestimation for Type 2 eyes at far distances under dim lighting, likely reflecting reduced visibility of vertical contours under those conditions. Together, these results show that even in fully 3D environments, small but systematic biases persist, shaped by lighting, distance, geometry, and the contrast structure of the eyeball.

The generalizability of our results is limited by several aspects. First, our Type 2 stimulus mimicked chimpanzee eyes in contrast but retained the blue-green iris hue of the live model, resembling species such as *Macaca fuscata* or *Ateles hybridus* and *A. fusciceps* (Meyer et al., 2013; Perea-García et al., 2022, 2024). However, many other primate species have amber-brown irises (like chimpanzees: Whitham et al., 2022b). Importantly, our stimulus does not aim to model ancestral human chromatic contrast. Rather, it isolates achromatic contrast relationships between iris and sclera. It is almost certain that ancestral human eyes did not exhibit this specific combination of dark sclera and low-melanin irises. Second, even though the sclerae of species such as mountain gorillas or chimpanzees have been described as ‘dark’ throughout the literature (Kobayashi & Kohshima, Kano et al., 2022b, 1997; Perea-García et al., 2019), we currently lack measurements of reflectivity from live primate eyeballs. Furthermore, the emphasis of recent reports on important variability at the species-level (e.g. Clark et al., 2023; Kano, 2023) complicates species-wide generalizations; there does not seem to be a unique, stable external eye appearance, especially for great ape species (Clark et al., 2023; Mayhew & Gómez, 2015).

In addition to the above, our sample size was relatively small (*N* = 20), which limits the extent to which these findings can be generalized beyond the present study. Furthermore, our participants were drawn from a WEIRD population, and cultural or experiential differences in gaze processing may influence performance. We emphasize that our results are informative for cross-species comparisons and do not bear on variation within extant human populations. In particular, they do not support the assumption that ‘uniformly white’ sclera are required for effective gaze signalling. Gaze-direction detection remained highly accurate across all conditions tested, indicating that a range of eye configurations is sufficient for this function. Moreover, the differences between our experimental stimuli were doubtlessly more extreme than typical variation in human external eye appearance. On this basis, our findings provide no support for the idea that variation in eye morphology across human populations leads to meaningful differences in communicative performance.

Also, our experiment only tested human participants, and the task did not introduce time restrictions for their response. If humans have a perceptual expertise for Type 1 eyes (as suggested by contrast-polarity studies like Ricciardelli et al., 2000), the disadvantage of Type 2 eyes in many combinations of factors in our study may be inflated. With regard to the lack of time constraints, the adaptive value of eye-gaze perception could be most pronounced in time-sensitive situations, so the general performance of our participants across all combinations of factors may be inflated. Lastly, although the temperature of our lighting is comparable across conditions, it was not chosen to closely align with ecologically relevant values. These factors may limit ecological validity.

Although our experiment does not address evolutionary processes directly, the pattern we observed fits broader models in which traits shaped for non-communicative functions later provide substrates for communicative refinement in specific ecologies. Comparable transitions are well documented: early feathers in non-avian dinosaurs appear to have evolved for insulation before being co-opted for display and flight (Benton et al., 2019), and red carotenoid pigmentation in birds likely originated in internal tissues before being relocated to external structures as social signals (Alonso-Alvarez et al., 2022). These cases exemplify exaptation (Gould & Vrba, 1982) and align with models in which latent morphological variation forms the basis for later functional elaboration under specific ecological and perceptual conditions (Erwin, 2021).

Under this view, selection for gaze signalling operates on pre-existing variation shaped by more basal pressures, such as photoprotection, rather than generating morphology de novo. As a result, different baseline eye appearances may channel subsequent communicative refinement in different directions. For example, in species with more heavily pigmented sclerae, further increases in pigmentation could enhance contrast with the iris under specific viewing conditions, whereas in species with lighter peri-iridal tissues, contrast may instead be achieved through depigmentation. Thus, the salience of Type 2 eyes in our study may reflect conditions in which tracking the eyes of conspecifics is advantageous (close range, shaded or dim lighting), while the salience of Type 1 eyes may reflect conditions favouring signalling over longer distances (Kano et al., 2022b). Under this conceptual framework, primate eye diversity may represent multiple viable substrates for gaze communication rather than the outcome of a single optimized solution.

Finally, adaptive explanations have dominated accounts of primate eye appearance, but our results warrant caution in treating visible eye morphology as a direct proxy for communicative function (cf. Caspar et al., 2021). The CEH and related models assume that human eye morphology evolved primarily for gaze signalling (Kobayashi & Kohshima, 1997; Tomasello et al., 2007; Yorzinski et al., 2021), yet this assumption remains untested. Ocular traits may instead reflect photoprotective demands, sexual selection, or drift (Perea‐garcía et al., 2025). Gaze signalling appears robust across diverse morphologies (Shaifei et al., 2026; Whitham et al., 2022a, 2022b), including our own stimuli, in which the iris–sclera contrast emphasized in the literature was similarly visible across morphologies. The difference in errors between stimuli we observed across viewing conditions would not meaningfully affect the functions proposed in recent revisions of the CEH (e.g. ‘group hunting at night’; Yorzinski et al., 2021), further weakening claims that specific morphological features are required for these behaviours. Claims about the centrality of eye-gaze following for human social cognition are often derived from relatively homogeneous (WEIRD) samples. Although cross-cultural work suggests that gaze following is widely present, its development and expression vary with early social experience and cultural context (Perea‐García et al., 2025). Future work should integrate species-specific ecologies, behaviours, and morphologies (Whitham et al., 2022a, 2022b) and examine non-human primates on their own terms rather than primarily as comparators for human functions.

The predictions of the CEH and its derivatives rest on the assumption that eye-gaze signalling is itself adaptive (Kobayashi & Kohshima, 2001; Tomasello et al., 2007). Our study targets a narrower claim, namely that Type 1 eyes confer an advantage for gaze signalling under conditions of reduced visibility. We do not test the adaptive value of gaze signalling, and our results speak only to its perceptual underpinnings. Whether such perceptual differences translate into consistent behavioural advantages, and ultimately into fitness-relevant outcomes, remain untested. Claims about the adaptive significance of eye-gaze following and its relationship with specific eye morphologies remain speculative.

In summary, our study challenges the view that human eye appearance excels at signalling, especially under conditions of poor visibility. Situating these results within broader models of trait evolution highlights that communicative capacities may emerge from the refinement of pre-existing variation, rather than from a uniquely optimized design. Progress in this line of research will depend on integrating species-specific ecological data, quantifying ocular traits across primates, and experimentally manipulating contrasts and cues in ways that reflect the environments in which gaze communication actually occurs.

## Supporting information

10.1017/ehs.2026.10058.sm001 Perea García et al. supplementary material Perea García et al. supplementary material

## Data Availability

All data on participants’ acuity are included in the dataset hosted in the online repository.

## Supplementary material.

The supplementary material for this article can be found at https://doi.org/10.1017/ehs.2026.10058.

## Research transparency and reproducibility interest

The *R* and *Matlab* code and data files used in the analyses and figure preparation can be accessed here: https://osf.io/2waud/overview?view_only=9eb66093ce9a46b781f4c211e94fd82d.

## Figure 1 Long description

Image A shows two individuals in a room with a grid of labels on the wall. One person is wearing a red-blue top and is seated at a table, facing the wall. The other person, wearing a green sweater, is seated at the table, gesturing towards the wall. Image B shows the same room divided by a yellow line, illustrating dim and bright lighting conditions. The model in the red-blue top is seated on a swiveling stool, facing the wall with the grid. A chart is visible on the wall in the bright condition. The room contains a table and a stool, with a radiator under a window in the dim condition.

Navigate back to Figure 1.

## Figure 3 Long description

The bar graph compares mean Manhattan distance error across different conditions. The horizontal axis has categories Bright and Dim. The vertical axis is labeled Mean Manhattan Distance Error, ranging from 0.0 to 2.5. The legend indicates Control (Type 1) and Experimental (Type 2) eye appearances, with far and near distances. Under Bright, Control Type 1 near is approximately 1.5 and far is around 1.6. Experimental Type 2 near is about 1.7 and far is close to 1.8. Under Dim, Control Type 1 near is approximately 1.7 and far is around 1.9. Experimental Type 2 near is about 2.0 and far is close to 2.2. Dim conditions generally show higher errors than Bright. Significant differences are marked with asterisks, indicating notable comparisons between conditions. The bars are vertical and whiskers depict standard error.

Navigate back to Figure 3.

## Table 1 Long description

The table reports mean Manhattan error and standard deviation for eight conditions combining brightness (bright or dim), distance (near or far), and type (Type 1 or Type 2). Mean error is lowest for dim near Type 2 at 1.43, followed closely by bright far Type 1 at 1.44 and bright near Type 1 at 1.54. Mean error is highest for dim far Type 2 at 2.16, with the next highest being dim far Type 1 at 1.87 and bright far Type 2 at 1.80. Across both types, far conditions generally have higher mean error than near conditions, especially under dim lighting. Standard deviation ranges from 1.35 to 1.83, with the smallest variability in bright near Type 1 at 1.35 and the largest in bright near Type 2 at 1.83. Differences between Type 1 and Type 2 vary by lighting and distance, so patterns should be interpreted as descriptive rather than causal.

Navigate back to Table 1.

## Table 2 Long description

The table reports pairwise differences from a reference condition, giving a log estimate, its exponentiated value, and a highest posterior density interval with a credibility flag and direction. Four comparisons are credibly greater than the reference: bright near Type 2 (log estimate 0.1903, exp estimate 1.21, interval 0.1012 to 0.2804), bright far Type 2 (0.2268, 1.26, 0.1371 to 0.3169), dim far Type 1 (0.2562, 1.29, 0.1061 to 0.4025), and dim far Type 2 (0.4146, 1.51, 0.3263 to 0.4996). The largest credible increase is dim far Type 2. Three comparisons are not credible because their intervals include zero: bright far Type 1 is slightly lower (log estimate minus 0.0087, exp estimate 0.99, interval minus 0.1692 to 0.1391), while bright near Type 1 (0.0573, 1.06, minus 0.0917 to 0.214) and dim near Type 1 (0.1582, 1.17, minus 0.0028 to 0.3016) trend higher. Overall, Type 2 conditions show consistent credible increases, while Type 1 results are mixed and more uncertain.

Navigate back to Table 2.
