# Supplementary material for: Gaze in context: non-human eyes can be more salient under ecologically relevant conditions
Source: Evol Hum Sci. 2026 Jun 19;8:e25. doi: 10.1017/ehs.2026.10058 (PMC13319479; doi:10.1017/ehs.2026.10058)
Supplement: Perea García et al. supplementary material [file S2513843X26100589sup001.docx]

**Supplementary Materials**

*Physical layout (S1)*
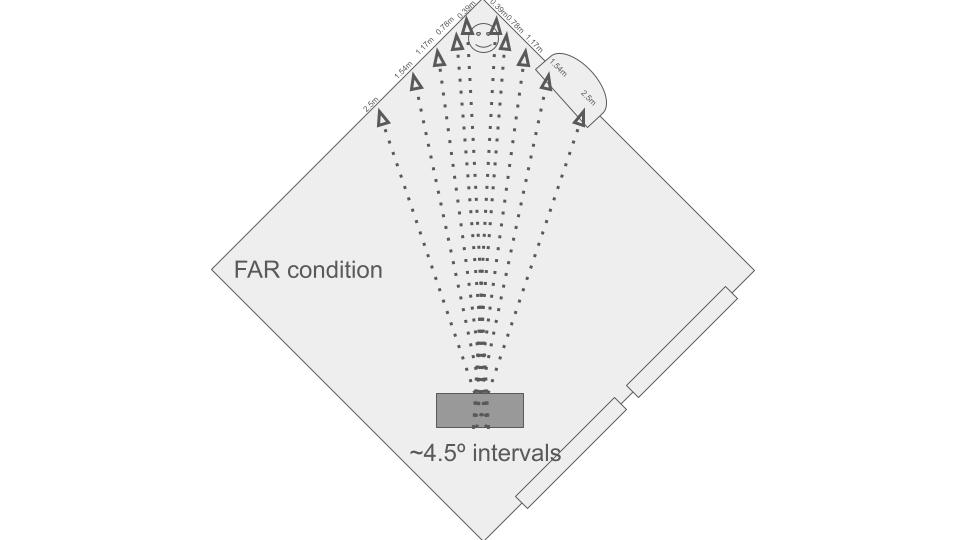

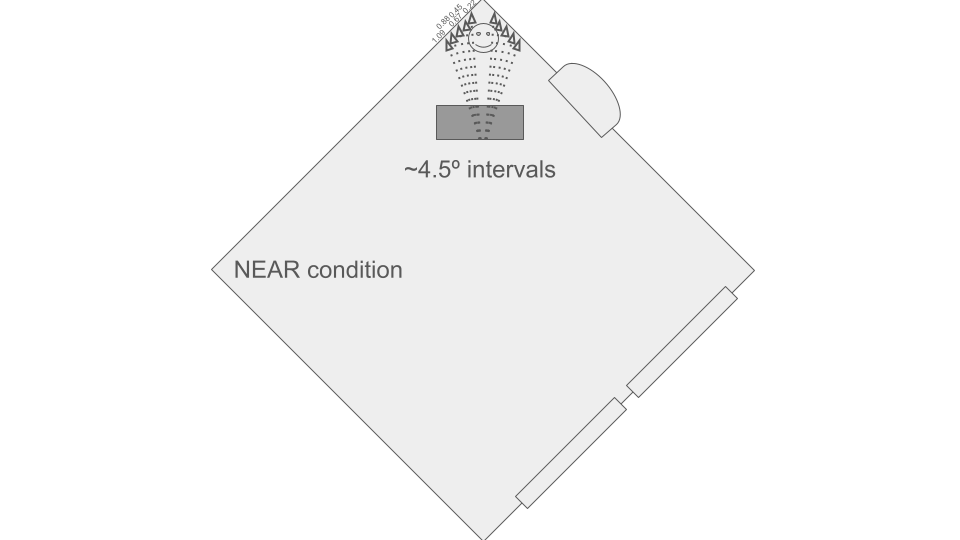


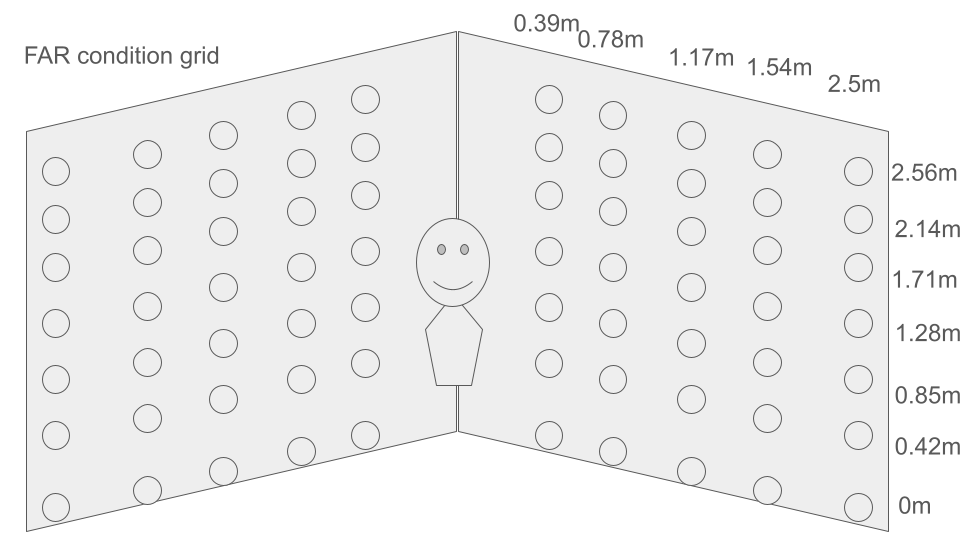


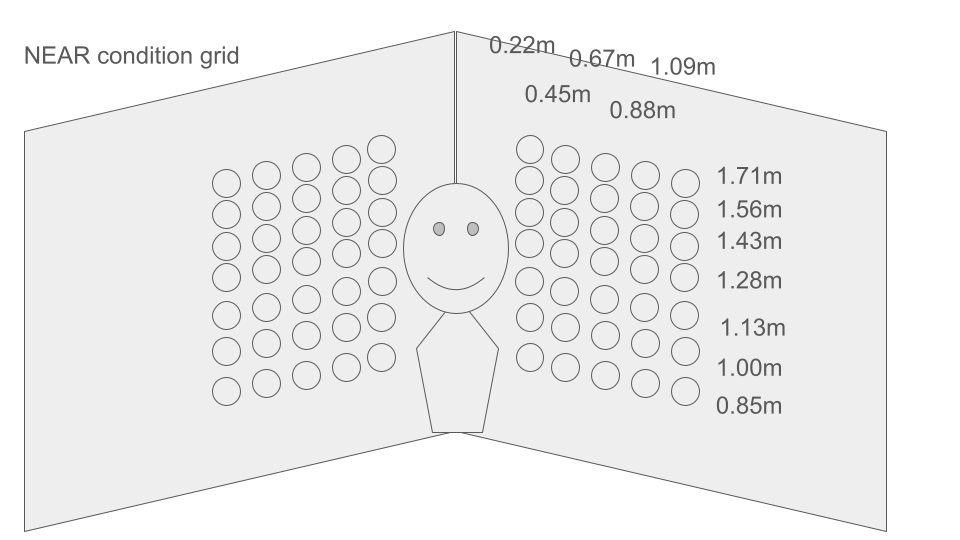


Figure S1.- Above: top-down view of the Far and Near distances. Below: frontal views of Far and Near distances, from the live model’s perspective.

*Lighting (S2)*
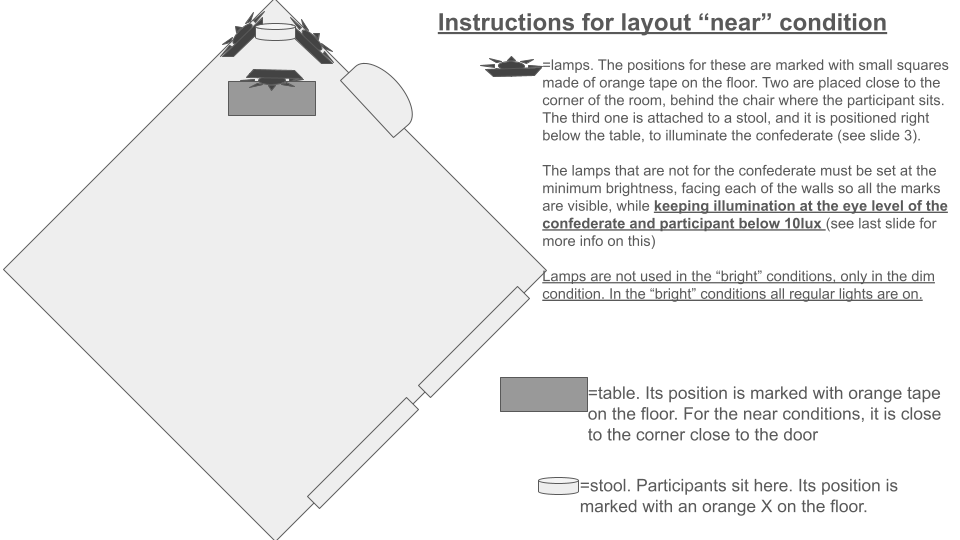


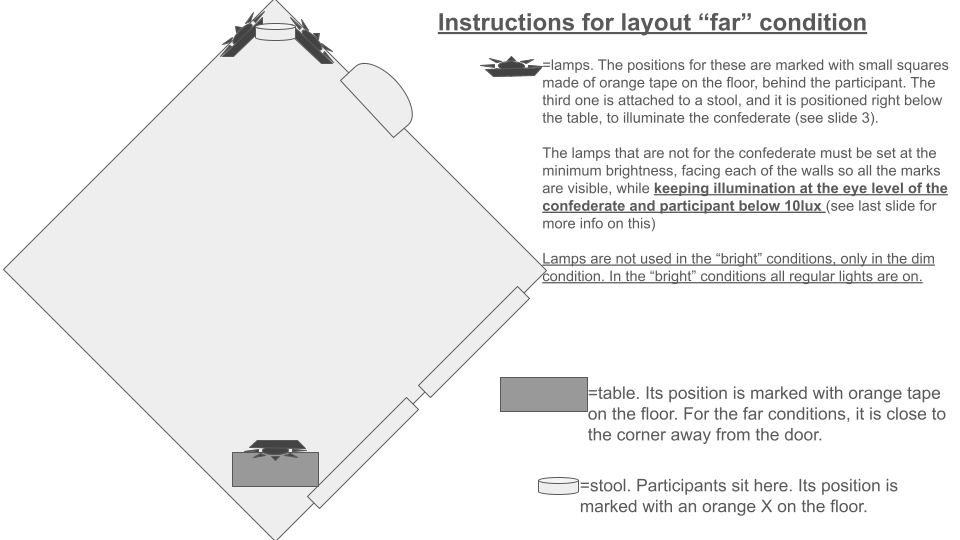


Figure S2.- Arrangement of the lighting setup in the Near (top) and Far (bottom) conditions. Specifications of the lamps used in the study: Item: LED Desk Lamp; LED Power: 23 W; Voltage: 220V~50 Hz; Adapter Output: 12 V == 2 A; USB Input: 5 V == 1 A; CCT (Correlated Color Temperature): 2800 K / 3500 K / 4200 K / 5000 K / 6000 K; CRI (Color Rendering Index): ≥ 80 Ra; Center Illuminance (40 cm): ≥ 1000 LUX; Material: Aluminum+Metal; Operating Temperature: -10°-40°C; Product Size: 660 × 193 × 753 mm; Net Weight: 1950 g

*S3 - results from model checking for training effects*

Table S3 - summary of results testing potential training effects

| **Section** | **Parameter** | **Estimate** | **Est. Error** | **l-95% CI** | **u-95% CI** | **Rhat** | **Bulk ESS** | **Tail ESS** |
| --- | --- | --- | --- | --- | --- | --- | --- | --- |
| Multilevel hyperparameters: participant, 20 levels | sd(Intercept) | 0.39 | 0.07 | 0.28 | 0.55 | 1.01 | 472 | 1178 |
| Regression coefficients | Intercept | 0.47 | 0.10 | 0.26 | 0.65 | 1.02 | 321 | 306 |
| Regression coefficients | session | 0.01 | 0.02 | -0.04 | 0.05 | 1.00 | 2224 | 2387 |
| Regression coefficients | trial | -0.00 | 0.00 | -0.00 | 0.00 | 1.00 | 9232 | 2458 |
| Further distributional parameters | shape | 140.24 | 82.75 | 48.35 | 360.35 | 1.00 | 1984 | 2530 |
| Further distributional parameters | zi | 0.00 | 0.00 | 0.00 | 0.00 | 1.00 | 1769 | 1026 |

*S4 - Heatmap - Error rate per referent*


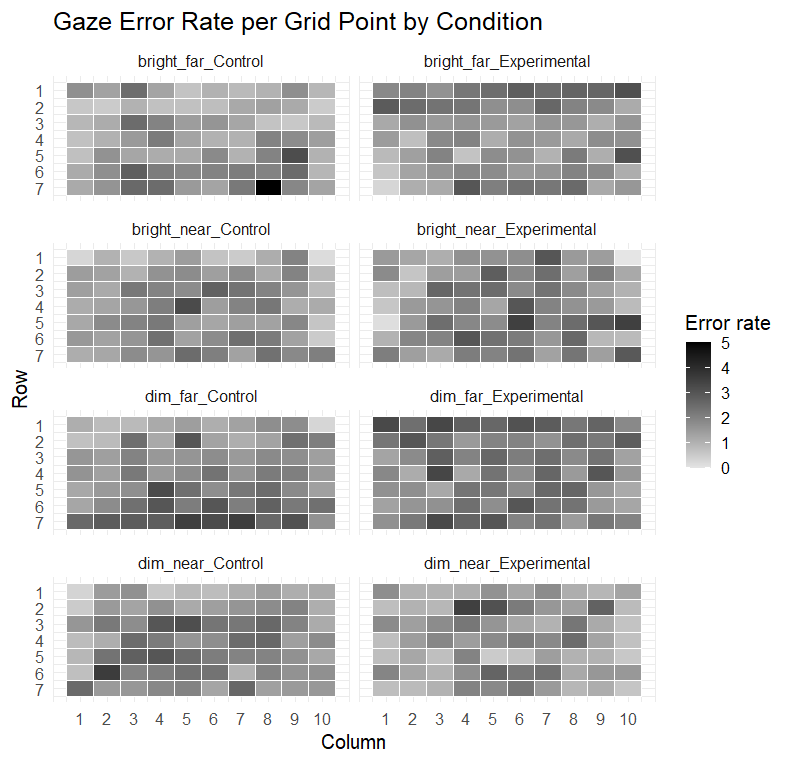


Figure S4.- Error rate by referent and combination of factors for both eye appearances.

*S5 - Mean Manhattan distance error by referent centrality*


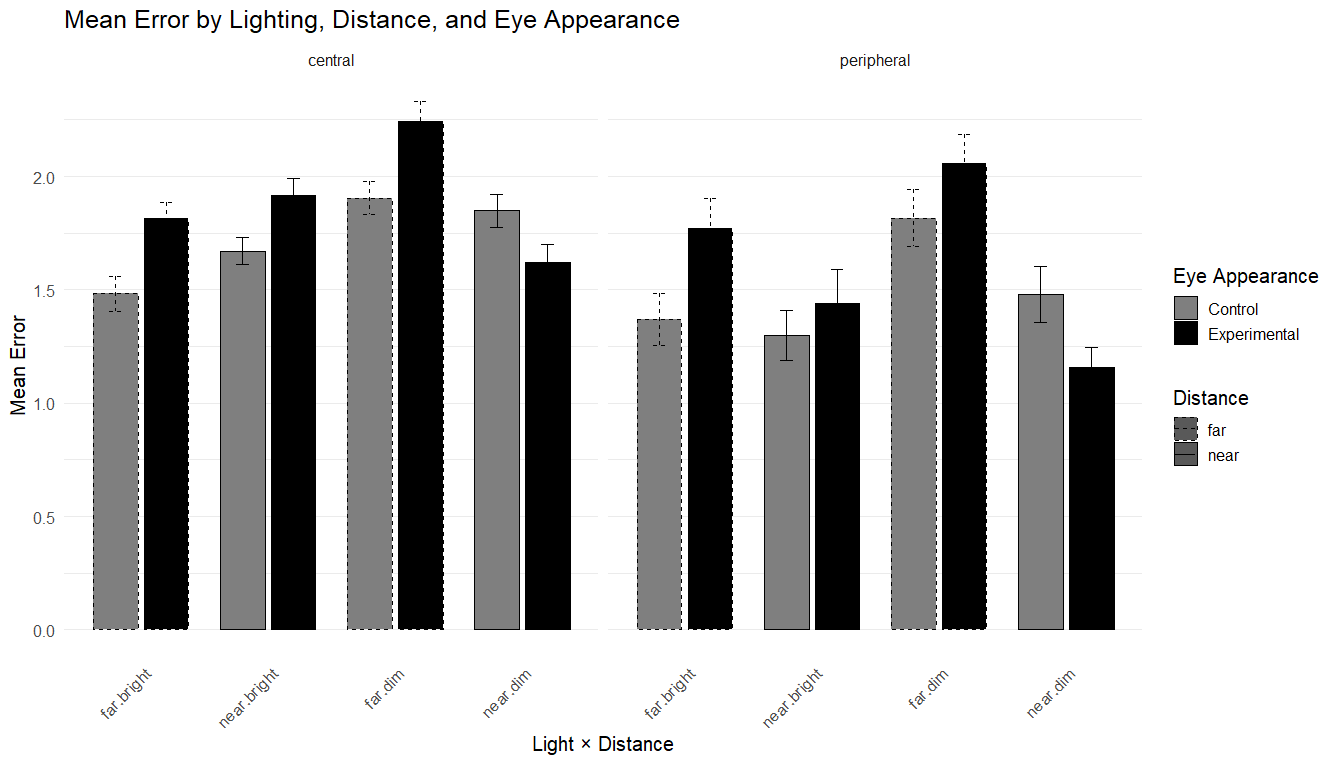


*Figure S5.- Bar plots for Mean Manhattan error by combination of factors, eye appearance, considering referent centrality*

*S6.- Gaze estimation biases*
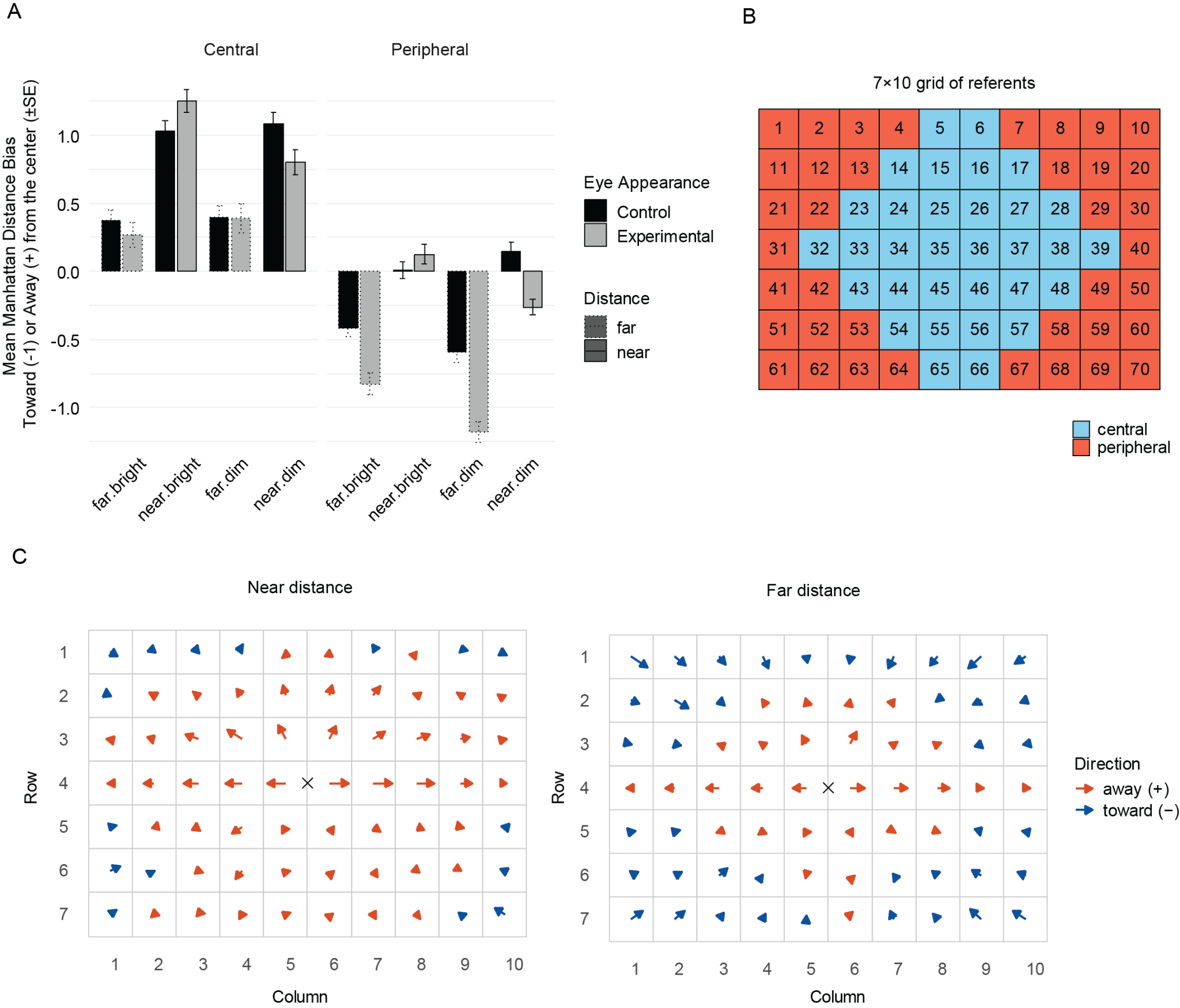


Figure S6. (A) Mean Manhattan distance bias as a function of viewing distance (near, far), lighting (dim, bright), referent centrality (central, peripheral), and eye appearance (control – Type 1; experimental – Type 2). The bias was calculated as the mean difference between the true referent’s distance from the screen center and the reported referent’s distance from the center. Positive values indicate that the reported location was farther from the center (away bias), whereas negative values indicate that it was closer to the center (toward bias). (B) Definition of central and peripheral referents within the 7 × 10 grid. (C) Visualization of the mean Manhattan distance bias in each cell of the 7 × 10 grid as a function of viewing distance (near, far). Orange arrows indicate a bias away from the center, and blue arrows indicate a bias toward the center. Arrow length corresponds to the magnitude of the bias.

*S7 - visual discriminability analyses*

*Pre-processing of photographs*

To model the visibility of different parts of the eye in both appearances, we first obtained high quality RAW photographs of both appearances in the exact same lighting, with the same camera settings. We then exported them as linear 16-bit TIFFs to preserve the proportional relationship between pixel values and scene luminance using RawTherapee (v.512). Images were exported with all tone curves, gamma correction, sharpening, etc. disabled. The output color space was set to RTv4_Wide, and black point compensation and highlight recovery were disabled. Regions of Interest (ROIs) were drawn over each part of the eye for both photographs according to the methods in Perea-García et al. (2024), treating the scleral as anterior peri-iridal tissues. The actual ROIs can be seen below in Figure S8. Because these TIFF values were linearly related to relative luminance, the measurements provided valid input for Weber contrast estimation, defined as the luminance difference between target and background divided by background luminance. ImageJ (v. 1.54p) was used to measure the brightness of the pupil, iris, sclera, and skin (Type 1 and Type 2), and of the scleral lens (Type 2). We used these measurements, together with the values for brightness and distance we recorded during the experiment for each combination of factors to estimate the visibility of each part of the eye, following Westland et al. (2006).

*CSF modeling*

Measurements of eye parts were taken for both [size](https://docs.google.com/spreadsheets/d/1RdeQnskPosbALqGxIaq2ULbce12x3OW8Elh4yu2OlAw/edit?gid=0#gid=0) (mm) and [brightness](https://docs.google.com/spreadsheets/d/1RdeQnskPosbALqGxIaq2ULbce12x3OW8Elh4yu2OlAw/edit?gid=1714941486#gid=1714941486) (linearized RGB from TIFFs). Left and right values were collapsed by averaging, so each Part × appearance has a single size and brightness summary.

[Angular sizes were calculated](https://docs.google.com/spreadsheets/d/1RdeQnskPosbALqGxIaq2ULbce12x3OW8Elh4yu2OlAw/edit?gid=632688596#gid=632688596) for the single feature (“detail”) that defines each pair (pupil, iris, sclera, or scleral lens) at two viewing distances (1300 mm, 5300 mm) using θ = 2 × arctan(d / (2D)), where d is that feature’s physical size and D is the viewing distance. Both radians (θ_rad) and degrees (θ_deg) were retained.

[Spatial frequencies were derived from the angular diameter of the detail.](https://docs.google.com/spreadsheets/d/1RdeQnskPosbALqGxIaq2ULbce12x3OW8Elh4yu2OlAw/edit?gid=632688596#gid=632688596) For each detail, cycles per degree was defined as f_cpd = 1/θ_deg, treating the detail’s angular extent as one cycle. Note that here “detail” refers to the smaller feature in each contrast pair that produces the visible boundary. Only cpd is used downstream.

[Pairs of interest were then constructed](https://docs.google.com/spreadsheets/d/1RdeQnskPosbALqGxIaq2ULbce12x3OW8Elh4yu2OlAw/edit?gid=1863374149#gid=1863374149) (e.g., pupil–iris, iris–sclera, scleral lens–sclera, scleral lens–skin, sclera–skin) were defined only to set which two regions form the Weber contrast. Geometry for CSF always uses the one detail feature listed above.

Relative brightness Y was computed for each part from linearized RGB using the [ROMM ProPhoto coefficients:
 Y = 0.2880 × R + 0.7119 × G + 0.0001 × B.](https://www.photo-lovers.org/pdf/color/romm.pdf)

Spaulding, K. E., Woolfe, G. J., & Giorgianni, E. J. (2000). Reference input/output medium metric rgb color encodings. In *Color Imaging Conference* (Vol. 1, p. 2).

The third-darkest gray ColorChecker patch was used as luminance reference. With known reflectance ρ_patch = 0.198, scaling factors were computed for each illumination scenario (6.5 lux dim, 500 lux bright):
 L_scale = E_lux × ρ_patch / (π × Y_patch).
 [Each part’s Y was multiplied by L_scale to yield luminance L_cd_m2.](https://docs.google.com/spreadsheets/d/1RdeQnskPosbALqGxIaq2ULbce12x3OW8Elh4yu2OlAw/edit?gid=707865955#gid=707865955)

[Weber contrasts were calculated between adjacent parts](https://docs.google.com/spreadsheets/d/1RdeQnskPosbALqGxIaq2ULbce12x3OW8Elh4yu2OlAw/edit?gid=14596318#gid=14596318). For each defined pair, contrast magnitude |C| = |(L_target − L_background)/L_background| was taken. The reciprocal S_weber = 1/|C| was computed to represent the detection threshold in the plots.

Luminance for CSF was taken from the detail region itself under each light condition (L_use for the feature used to compute θ_deg and f_cpd).

A luminance-dependent CSF model (Westland et al. 2006) was applied:
 CSF(f, w, L) = a·f·exp(−b·f)·√(1 + c·exp(b·f)),
 where f is spatial frequency (cycles/deg), w is the detail’s angular size (deg), and L is the detail luminance (cd/m²). The parameters a, b, and c depend on L and w.

For each pair × distance × illumination, CSF curves were generated across a log-spaced frequency grid using w = θ_deg of the detail and the corresponding luminance L_use.

At the detail frequency f_cpd, the predicted CSF value (CSF_at_f) was extracted. The Weber-based sensitivity equivalent is S_weber = 1/|C| from the pair’s luminance contrast. A detectability ratio DR = CSF_at_f / S_weber was computed for each condition. CSF panels show CSF(f) with a vertical line at f_cpd, a point at CSF_at_f, and a horizontal line at S_weber.

*S8 - ROIs used to measure brightness*


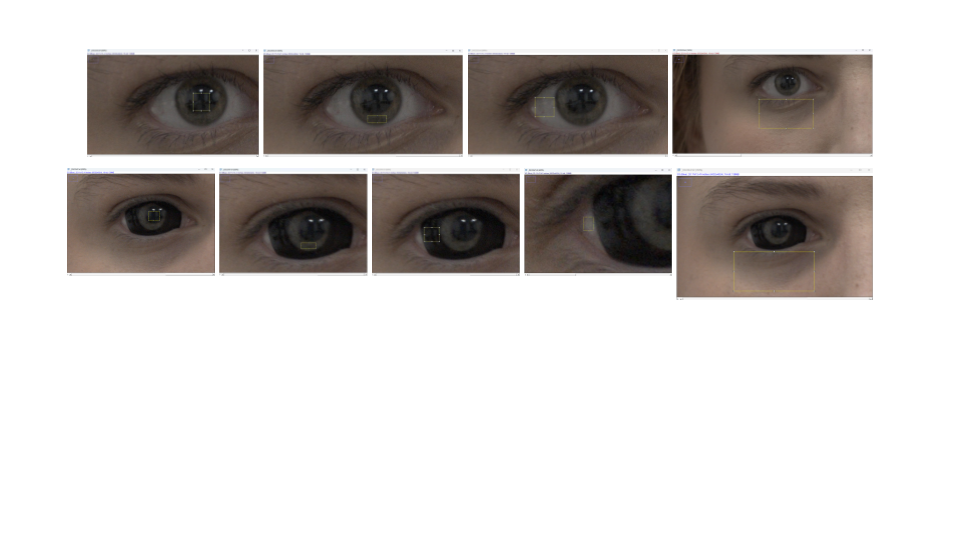


*Figure S8.- ROIs used in ImageJ to measure brightness of different parts of the eye in both eye appearances*

*S9- CSF models*
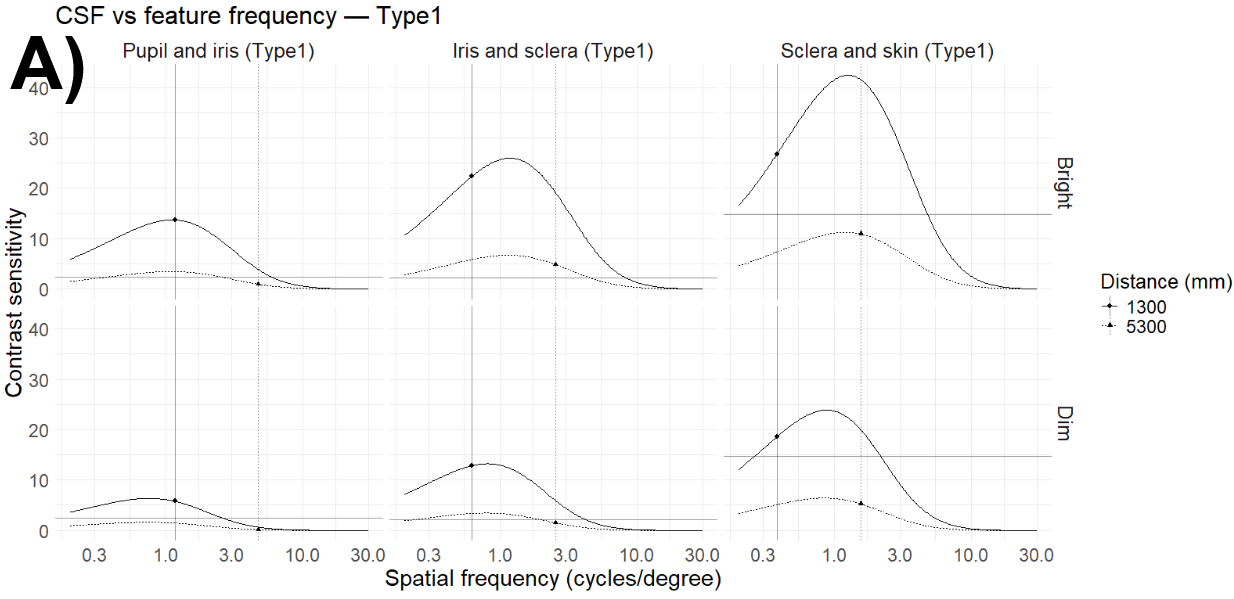


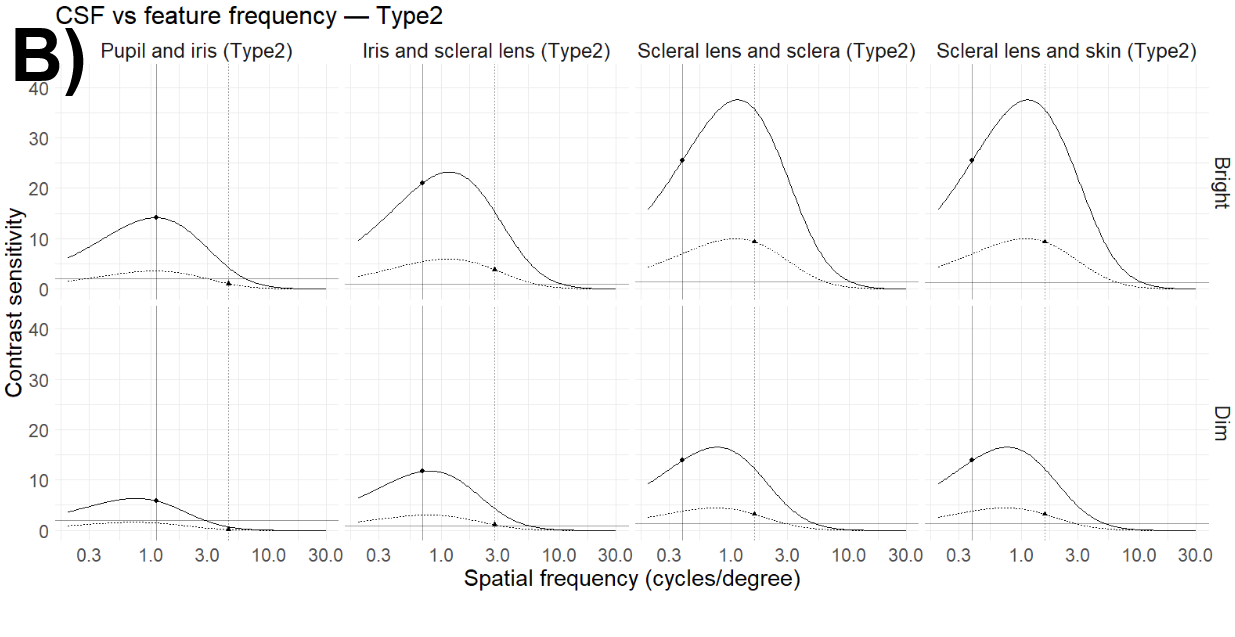


*Figure S9.- Contrast sensitivity function (CSF) in each combination of factors, by appearance. Continuous lines depict the function in the near distance, and discontinuous lines at the far distance. The horizontal line demarcates the threshold for detectability. In the key combination of factors dim near, A) shows that only irido-scleral contrast is visible for the Type 1 stimulus. B) shows that the Type 2 stimulus has a discernible contrast between iris and scleral lens, but also between scleral lens and underlying sclera, and between the scleral lens and surrounding skin.*

S10.- Illustration of decomposition in different spatial frequencies
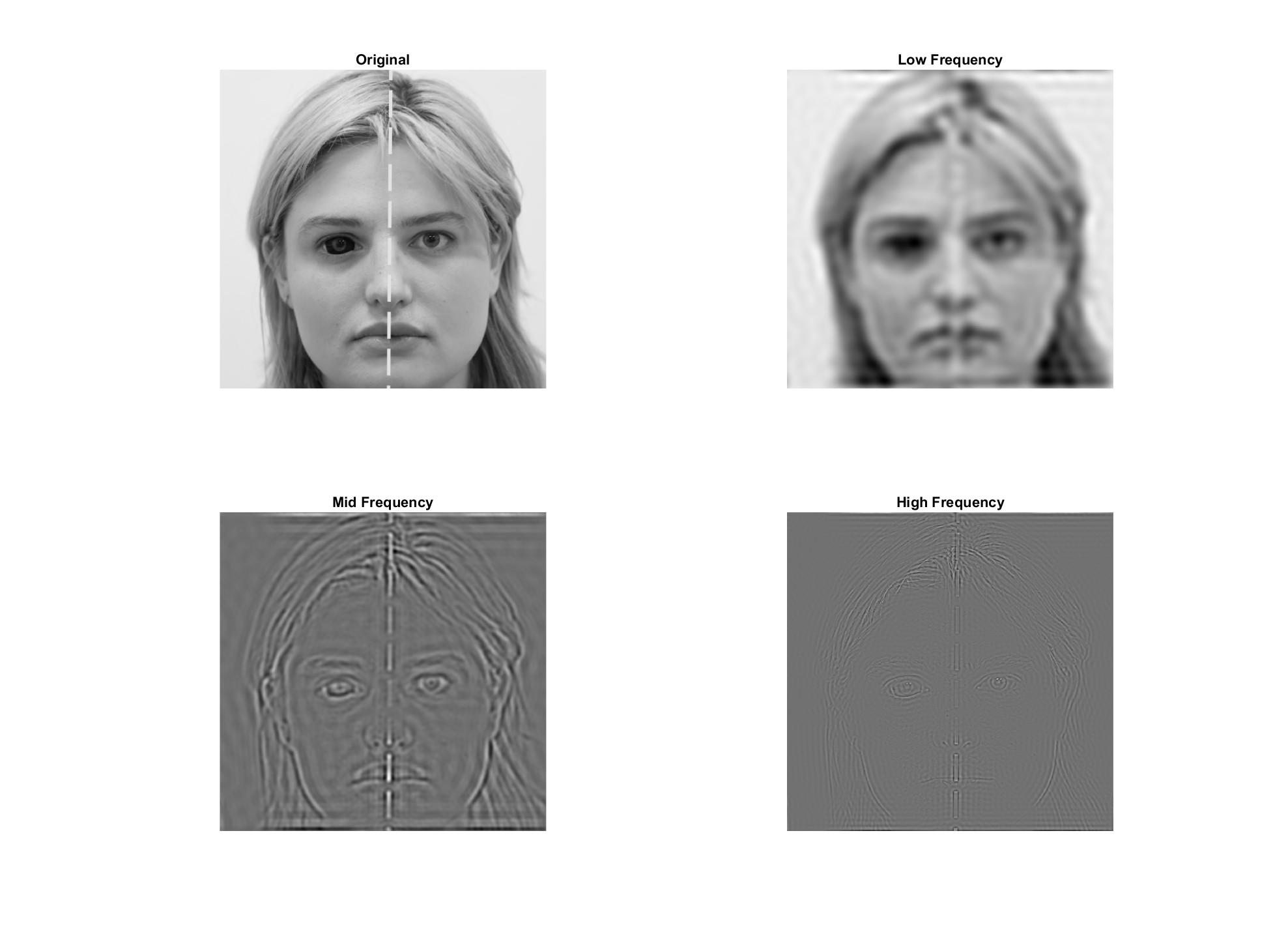


*Figure S10. Decomposition of an image into low-, middle-, and high-frequency spatial components. The original grayscale image (1400*1400 pixels leftmost panel) was decomposed using a 2D Fourier transform and frequency-selective circular masks to isolate different spatial frequency bands. The low-frequency component retains coarse luminance gradients and global shape (cutoff radius ≤ 20 pixels), the middle-frequency component captures intermediate spatial features (radius 20–60 pixels), and the high-frequency component preserves fine details and edges (radius > 60 pixels). These cutoff values are expressed in pixels and are arbitrary in the absence of precise viewing geometry, but serve to qualitatively illustrate the relative contribution of different spatial frequency ranges to image perception. The spatial-domain images were reconstructed using inverse Fourier transforms.*
